# Supplementary material for: How global DNA unwinding causes non-uniform stress distribution and melting of DNA
Source: PLoS One. 2020 May 15;15(5):e0232976. doi: 10.1371/journal.pone.0232976 (PMC7228070; doi:10.1371/journal.pone.0232976)
Supplement: S1 File — (PDF) [file pone.0232976.s001.pdf]

Supporting Information

How global DNA unwinding causes non-uniform stress distribution and melting of DNA

Korbinian Liebl and Martin Zacharias

February 18, 2020

Contents

1 Convergence of Umbrella Sampling free energy simulations 4

2 Calculation of Stiffness and Covariance Matrices 4

3 Elastic Energy Profiles 4

4 Ising model 33

5 Calculation of Geometric parameters through Rigid-Body Transformation 33

List of Figures

S1 Umbrella Sampling - Convergence - at 4
S2 Umbrella Sampling - Convergence -gc 5
S3 Stiffness matrix -at 5
S4 Stiffness matrix -gc 6
S5 Covariance - at 6
S6 Covariance - Restrained Case - at 7
S7 Covariance - gc 8
S8 Covariance - Restrained Case -gc 9
S9 Elastic Energy - at - unrestrained simulation 10
S10 Elastic Energy - gc - unrestrained simulation 11
S11 Elastic Energy - at - window 1 12
S12 Elastic Energy - at - window 2 13
S13 Elastic Energy - at - window 3 14
S14 Elastic Energy - at - window 4 15
S15 Elastic Energy - at - window 5 16
S16 Elastic Energy - at - window 6 17
S17 Elastic Energy - at - window 7 18
S18 Elastic Energy - at - window 8 19
S19 Elastic Energy - at - window 9 20
S20 Elastic Energy - at - window 10 21
S21 Elastic Energy - at - window 10 22
S22 Elastic Energy - gc - window 2 23
S23 Elastic Energy - gc - window 3 24
S24 Elastic Energy - gc - window 4 25
S25 Elastic Energy - gc - window 5 26
S26 Elastic Energy - gc - window 6 27
S27 Elastic Energy - gc - window 7 28
S28 Elastic Energy - gc - window 8 29
S29 Elastic Energy - gc - window 9 30
S30 Elastic Energy - gc - window 10 31
S31 Elastic Energy - gc - window 11 32

S32 Estimation of bubble-length from the Ising model 32

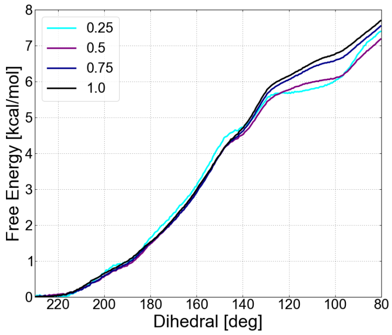

Figure S1: Free Energy profiles obtained from subintervals of the Umbrella Sampling simulations (for the at-sequence).

1 Convergence of Umbrella Sampling free energy simulations

The convergence of our Umbrella Sampling Simulations was checked through splitting the trajectories in subintervals (corresponding to the first 25 %, 50 %, 75 % or 100 % of the Umbrella sampling trajectories). The resulting PMFs indicate only little changes of less than 0.5 kcal/mol especially between using 75 % or 100 % of the Umbrella sampling data (Figure S1, S2).

2 Calculation of Stiffness and Covariance Matrices

Computed stiffness matrices are shown in Figure S3 and S4. Covariance matrices under relaxed and restrained cases are depicted in Figure S5-S8.

3 Elastic Energy Profiles

For the unrestrained simulation as wells as every Umbrella window we calculated the average elastic energy of all central 43 base-pair steps by using the harmonic model as explained in the previous section. The energy was projected on every step by the sum of its diagonal and half of the coupling terms. As shown in Figure S9 and S10, energy equipartitioning is accurately obtained in the unrestrained simulation. The profiles of the Umbrella Sampling windows are shown in Figurere S20-S31.

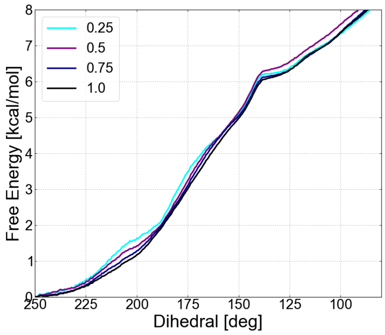

Figure S2: Free Energy profiles obtained from subintervals of the Umbrella Sampling simulations (for the gc-sequence).

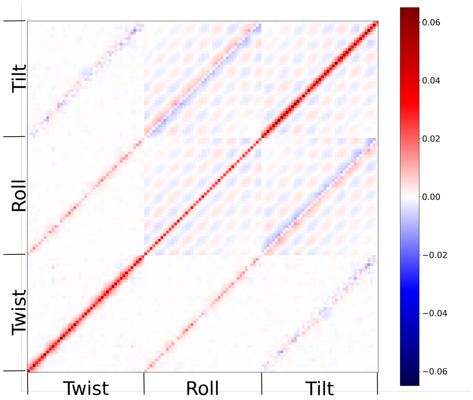

Figure S3: Computed stiffness-matrix for at-sequence, in units of $\frac{kcal}{mol \cdot deg^2}$. Note, the matrix is presented with the twist parameter of each base pair step along the sequence, followed by roll and tilt for each base pair step on the x- and y-axis. Each colored dot represents a stiffness force constant (color-coded and in units of $\frac{kcal}{mol \cdot deg^2}$). Only diagonal stiffness parameters and nearest-neighbor parameters deviate significantly from zero whereas the white areas represent near-zero stiffness entries (coupling beyond nearest neighbors is weak).

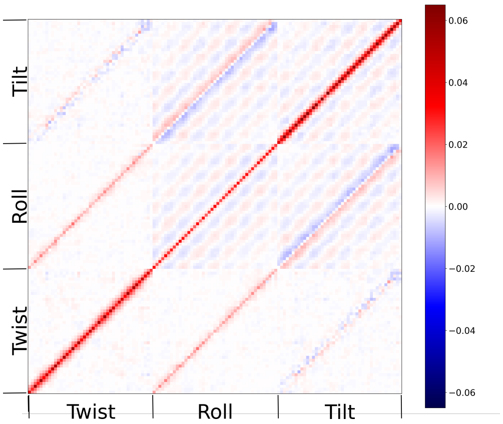

Figure S4: Computed stiffness-matrix for gc-sequence, in units of $\frac{kcal}{mol \cdot deg^2}$. The order of matrix entries is the same as explained in legend of Figure S3.

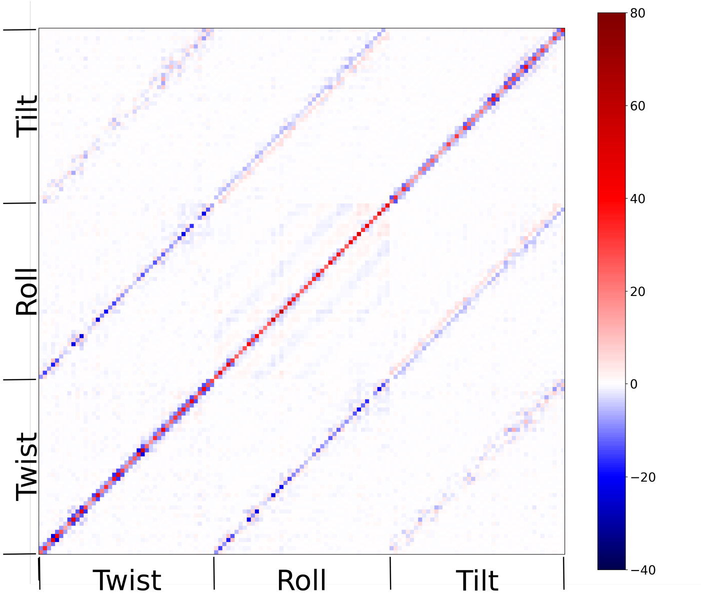

Figure S5: Covariance matrix obtained from unrestrained MD simulation of the AT-sequence, in units of deg^2. The order of matrix entries is the same as explained in legend of Figure S3

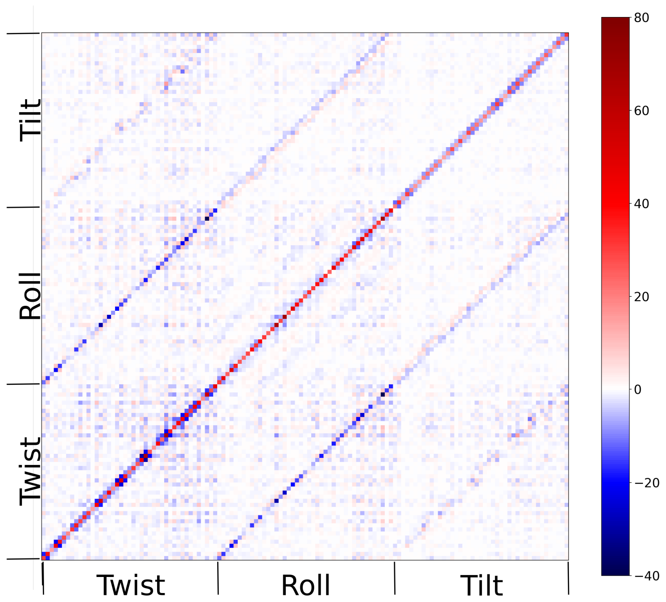

Figure S6: Covariance matrix obtained for the AT-sequence under high torsional stress ($0.054 \leq \sigma \leq 0.067$), in units of deg^2. The order of matrix entries is the same as explained in legend of Figurere S3. An increased correlation between helical parameters of distant base pair steps is observed compared to the unrestrained case.

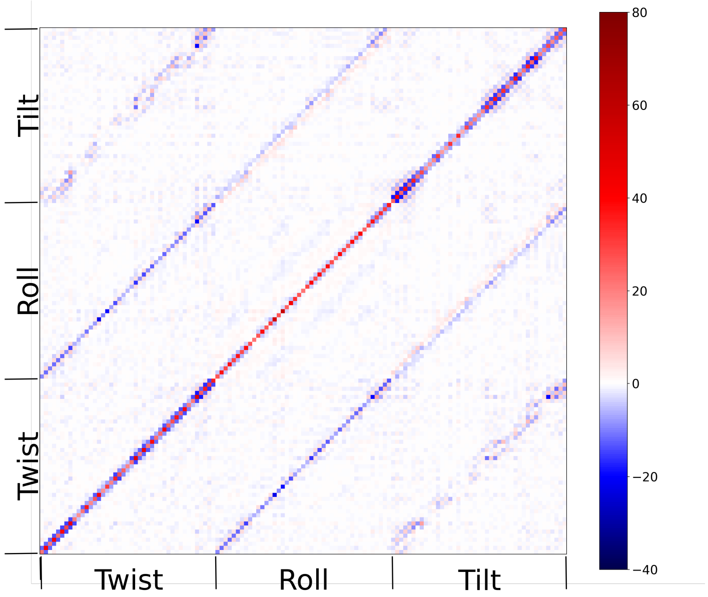

Figure S7: Covariance matrix obtained from unrestrained MD simulation of the GC-sequence, in units of deg^2. The order of matrix entries is the same as explained in legend of Figure S3

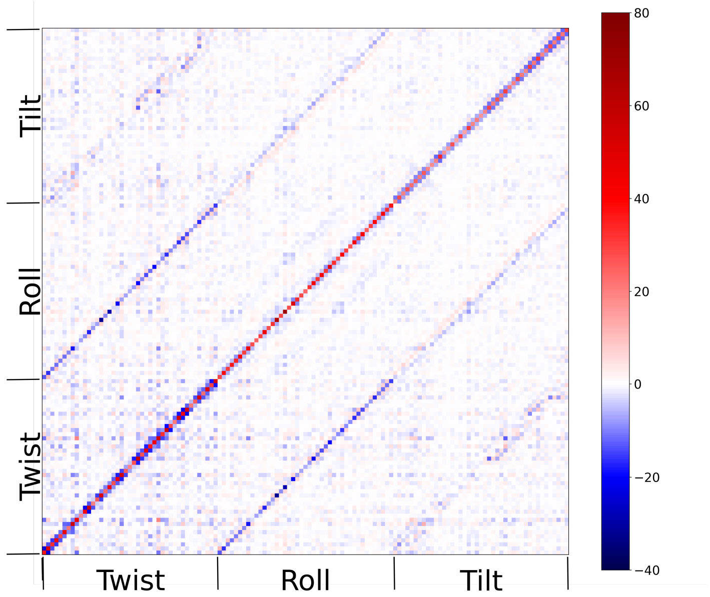

Figure S8: Covariance matrix obtained for the GC-sequence under high torsional stress ($0.053 \leq \sigma \leq 0.067$), in units of deg^2. The order of matrix entries is the same as explained in legend of Figure S3. An increased correlation between helical parameters of distant base pair steps is observed compared to the unrestrained case.

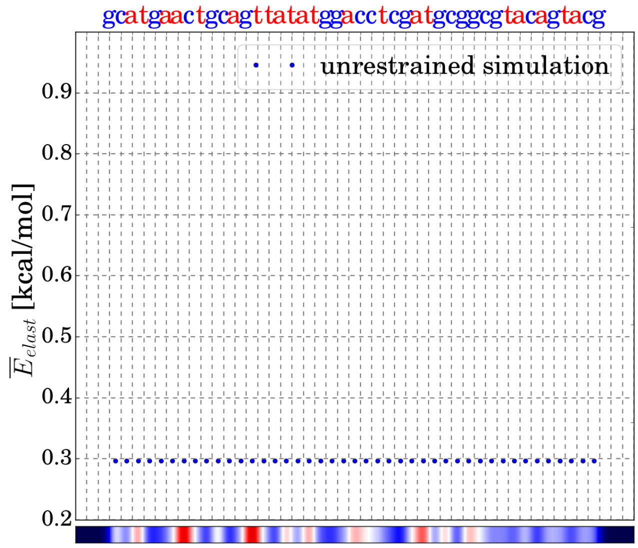

Figure S9: Average elastic energy for all central 43 base-pair steps for the at-sequence in the unrestrained case (no external torsional stress). Each vertical dotted line represents one base pair and the blue dots correspond to the average elastic deformation energy at each base pair step. Colormaps at the bottom reflect the calculated local twist-stiffness'at each base pair step (stiffness force constant from 0.01 (blue) to 0.045 (red) $kcal \cdot mol^{-1} \cdot deg^{-2}$).

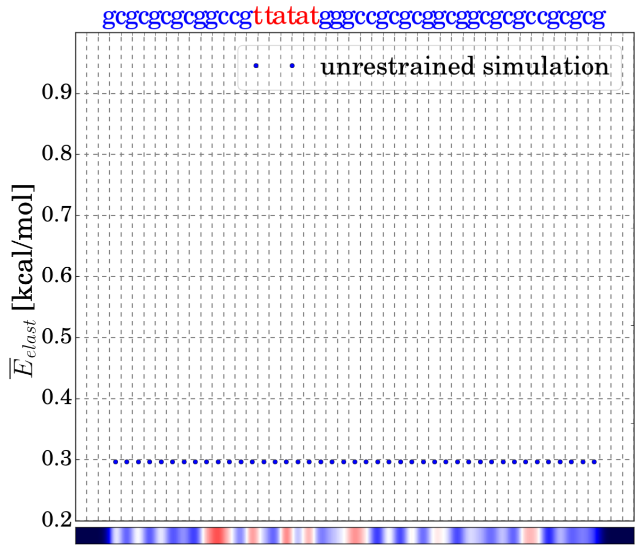

Figure S10: Average elastic energy for all central 43 base-pair steps for the at-sequence simulated in the unrestrained case (no external torsional stress). Colormaps at the bottom reflect the calculated local twist-stiffness'at each base pair step (stiffness force constant from 0.01 (blue) to 0.045 (red) $kcal \cdot mol^{-1} \cdot deg^{-2}$).

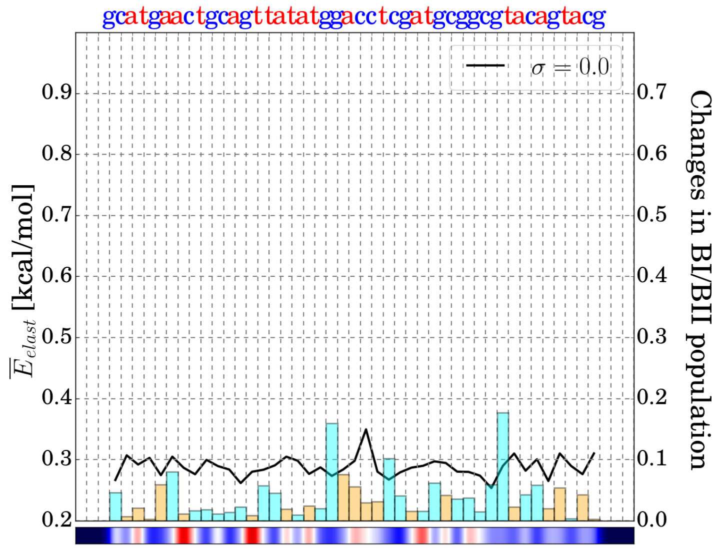

Figure S11: Average elastic energy (black line) for all central 43 base-pair steps for the at-sequence simulated in the first Umbrella window (minimal torsional stress). Changes in the backbone substate population of the two dominant BI and BII states are given as relative changes with respect to the unrestrained simulation case. Cyan bars indicate an average increase in BI state at the base pair position whereas orange bars reflect an increase in the average BII population. Colormaps at the bottom reflect the calculated local twist-stiffness'at each base pair step (stiffness force constant from 0.01 (blue) to 0.045 (red) $kcal \cdot mol^{-1} \cdot deg^{-2}$).

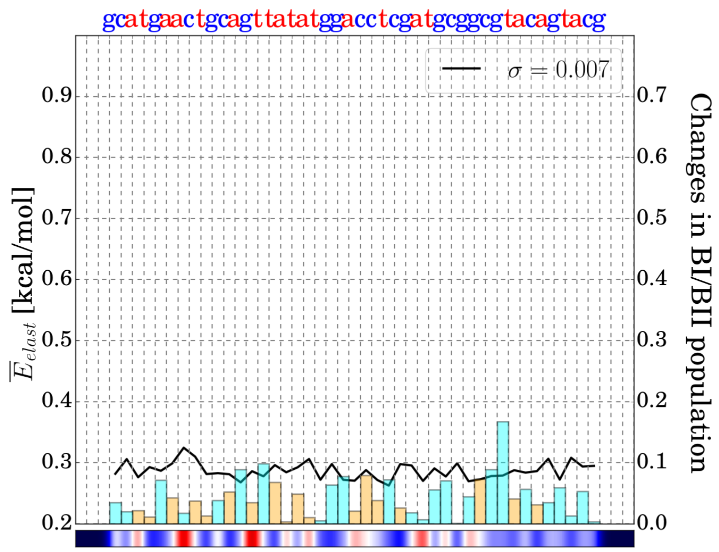

Figure S12: Average elastic energy (black line) for all central 43 base-pair steps for the at-sequence simulated in the second Umbrella window. The plot was generated in the same way as Figure S11.

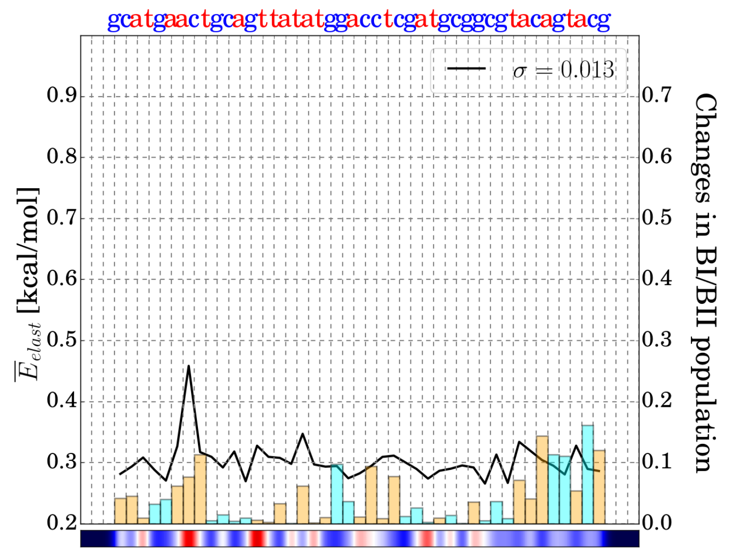

Figure S13: Average elastic energy for all central 43 base-pair steps for the at-sequence simulated in the third Umbrella window. The plot was generated in the same way as Figure S11.

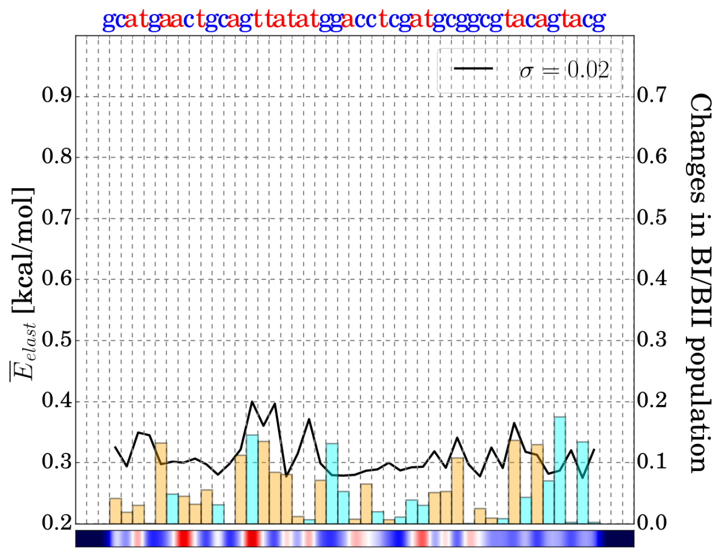

Figure S14: Average elastic energy for all central 43 base-pair steps for the at-sequence simulated in the fourth Umbrella window. The plot was generated in the same way as Figure S11.

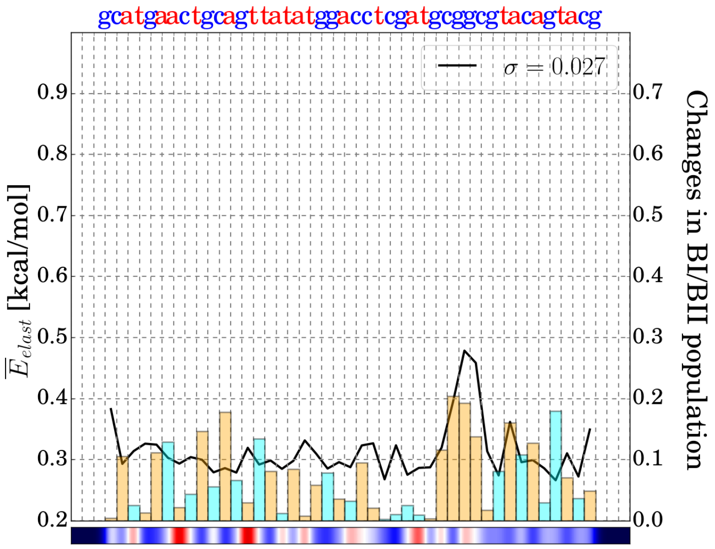

Figure S15: Average elastic energy for all central 43 base-pair steps for the at-sequence simulated in the 5th Umbrella window. The plot was generated in the same way as Figure S11.

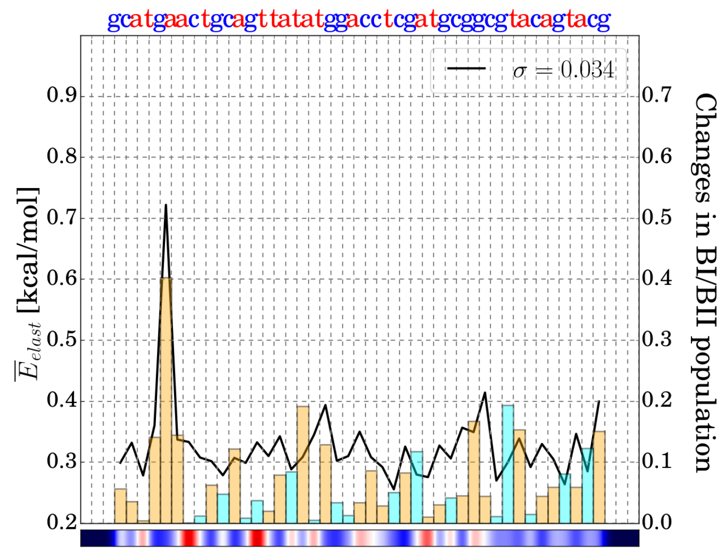

Figure S16: Average elastic energy for all central 43 base-pair steps for the at-sequence simulated in the 6th Umbrella window. The plot was generated in the same way as Figure S11.

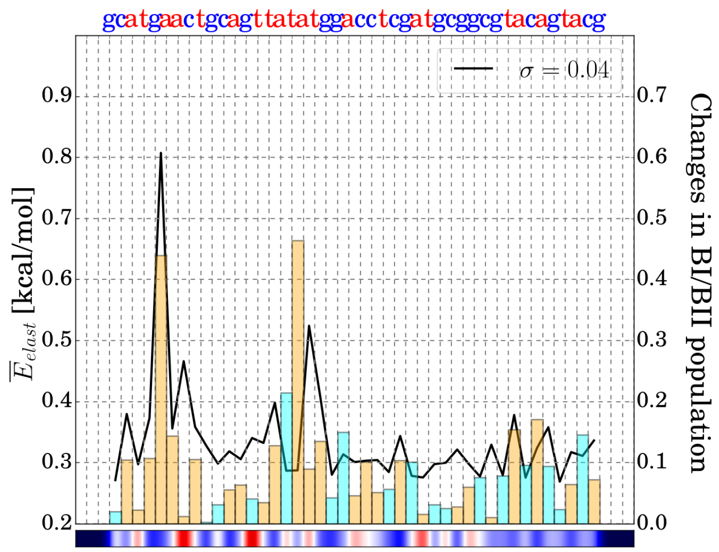

Figure S17: Average elastic energy for all central 43 base-pair steps for the at-sequence simulated in the 7th Umbrella window. The plot was generated in the same way as Figure S11.

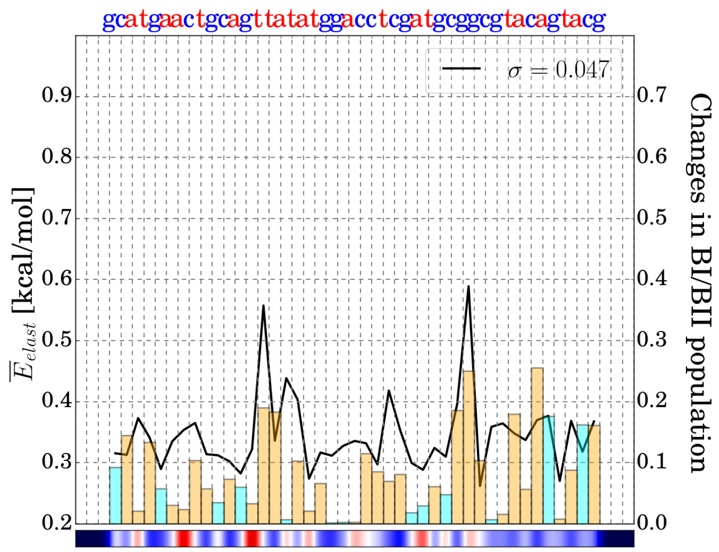

Figure S18: Average elastic energy for all central 43 base-pair steps for the at-sequence simulated in the 8th Umbrella window. The plot was generated in the same way as Figure S11.

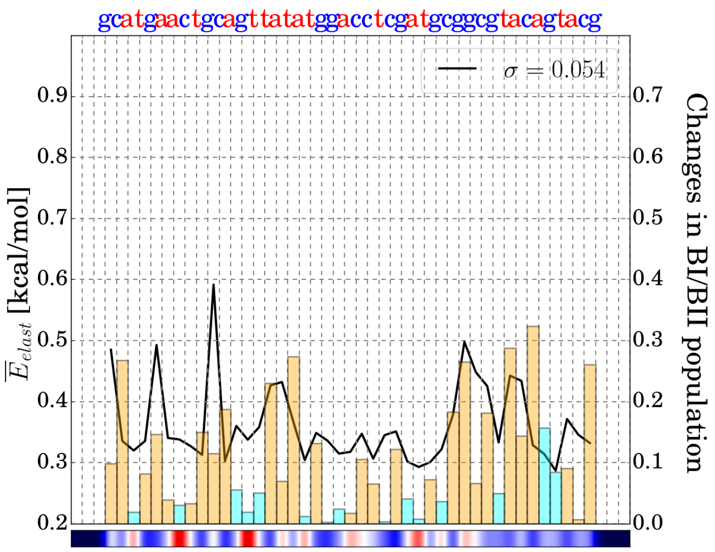

Figure S19: Average elastic energy for all central 43 base-pair steps for the at-sequence simulated in the 9th Umbrella window. The plot was generated in the same way as Figure S11.

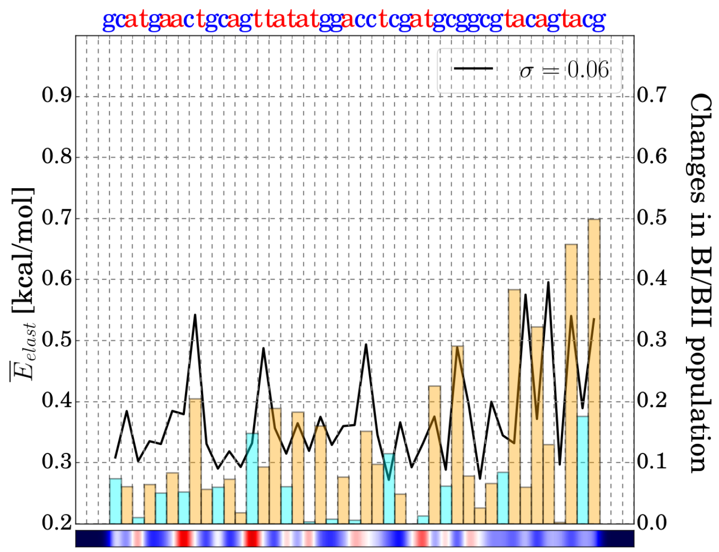

Figure S20: Average elastic energy for all central 43 base-pair steps for the at-sequence simulated in the 10th Umbrella window. The plot was generated in the same way as Figure S11.

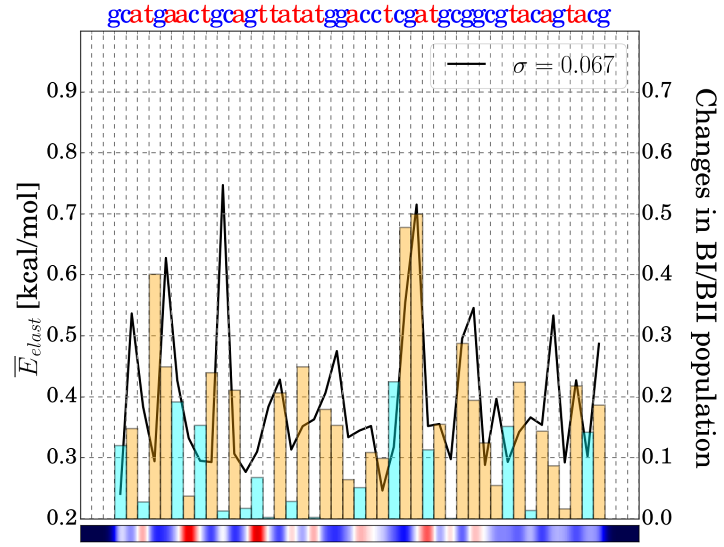

Figure S21: Average elastic energy for all central 43 base-pair steps for the at-sequence simulated in the 11th Umbrella window. The plot was generated in the same way as Figure S11.

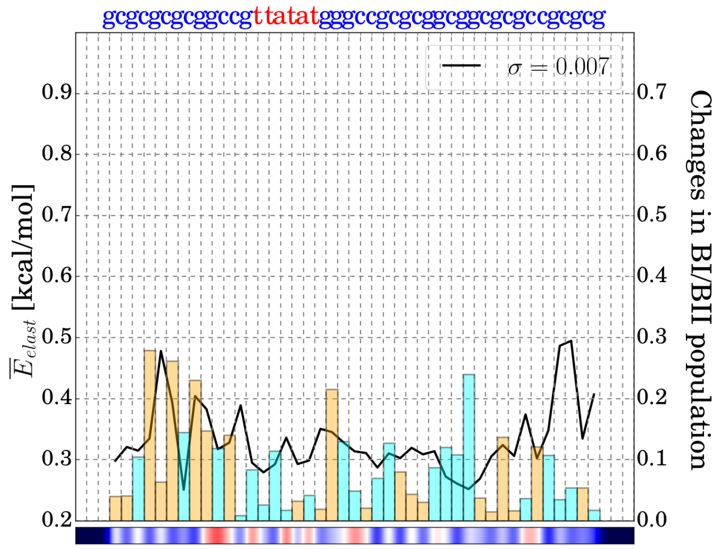

Figure S22: Average elastic energy for all central 43 base-pair steps for the gc-sequence simulated in the first Umbrella window (minimal torsional stress). Changes in the backbone population are given as relative changes. Cyan bars indicate increase in BI, orange bars increase in BII population. Bottom colorbar indicates local twist-stiffnes, see Figure S11.

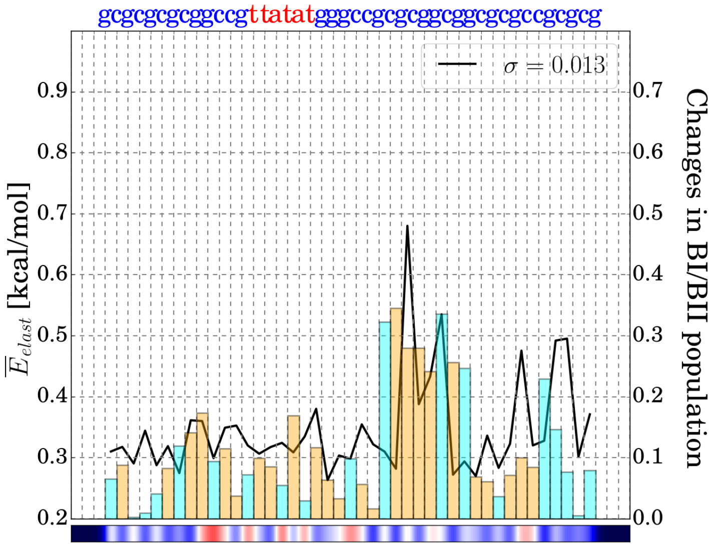

Figure S23: Average elastic energy for all central 43 base-pair steps for the gc-sequence simulated in the second Umbrella window. The plot was generated in the same way as Figure S22.

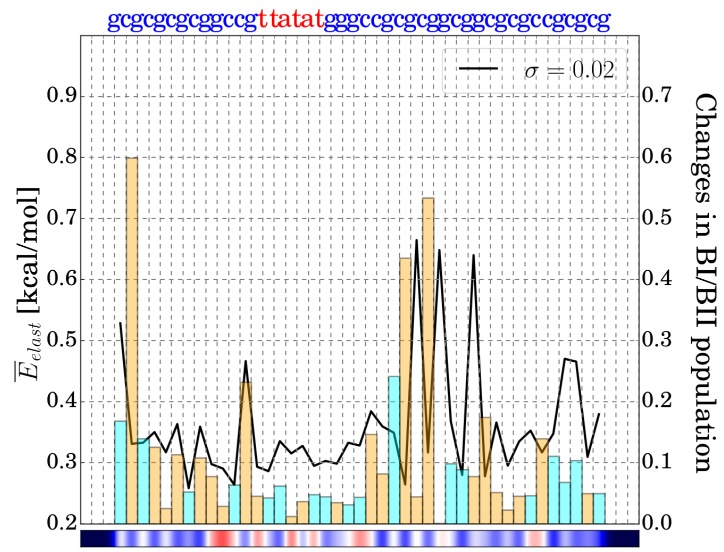

Figure S24: Average elastic energy for all central 43 base-pair steps for the gc-sequence simulated in the third Umbrella window. The plot was generated in the same way as Figure S22.

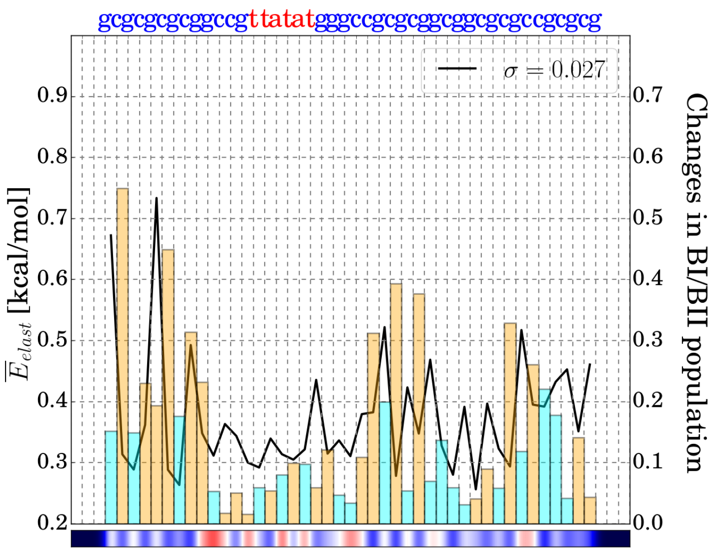

Figure S25: Average elastic energy for all central 43 base-pair steps for the gc-sequence simulated in the fourth Umbrella window. The plot was generated in the same way as Figure S22.

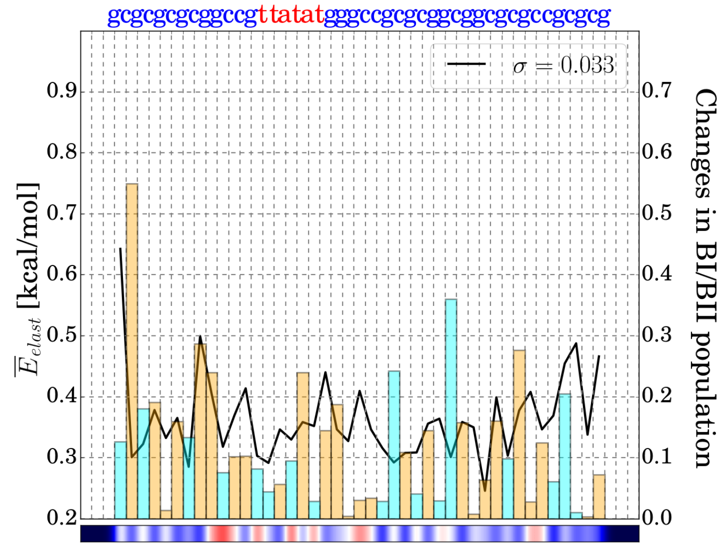

Figure S26: Average elastic energy for all central 43 base-pair steps for the gc-sequence simulated in the 5th Umbrella window. The plot was generated in the same way as Figure S22.

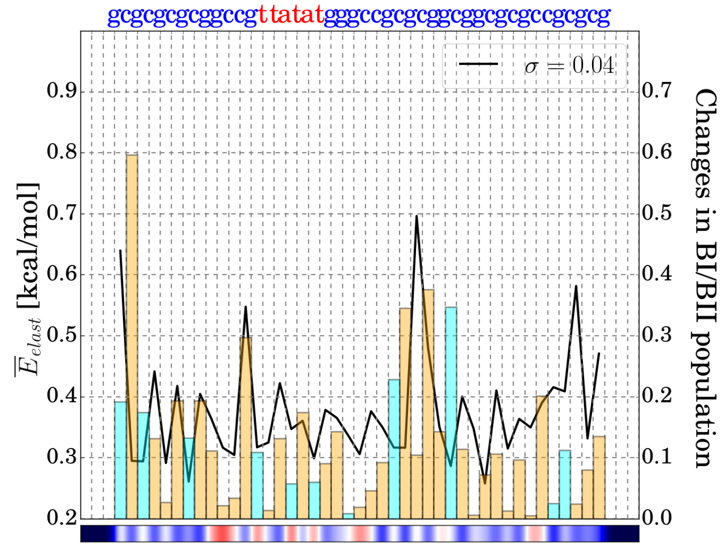

Figure S27: Average elastic energy for all central 43 base-pair steps for the gc-sequence simulated in the 6th Umbrella window. The plot was generated in the same way as Figure S22.

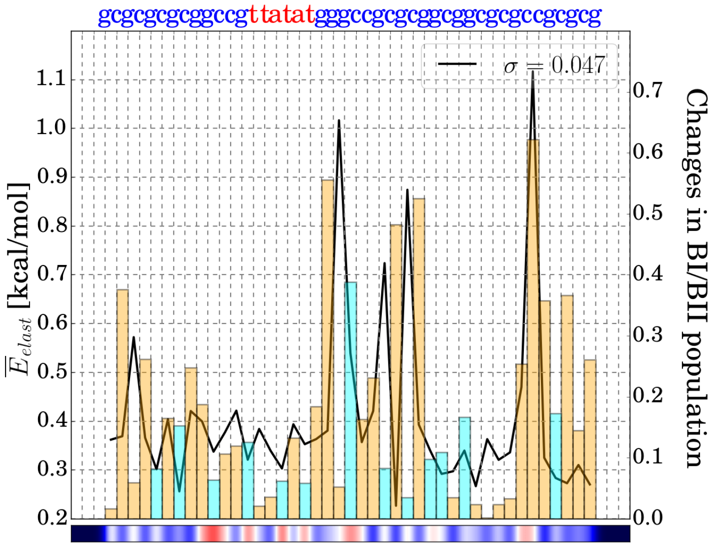

Figure S28: Average elastic energy for all central 43 base-pair steps for the gc-sequence simulated in the 7th Umbrella window. The plot was generated in the same way as Figure S22.

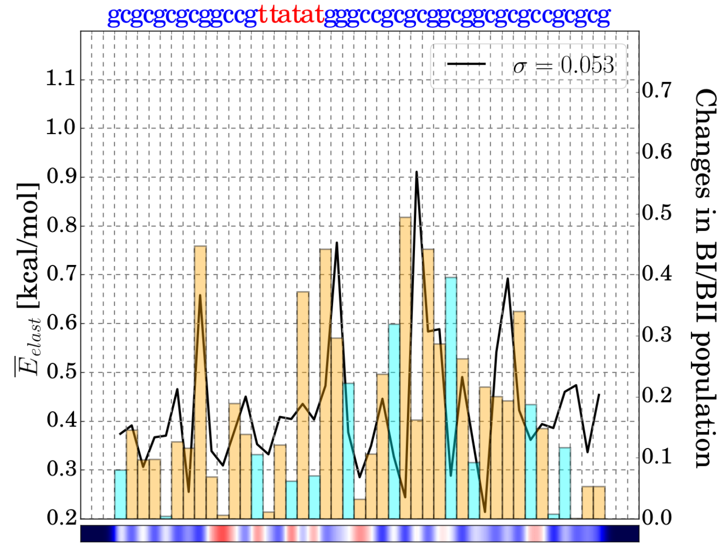

Figure S29: Average elastic energy for all central 43 base-pair steps for the gc-sequence simulated in the 8th Umbrella window. The plot was generated in the same way as Figure S22.

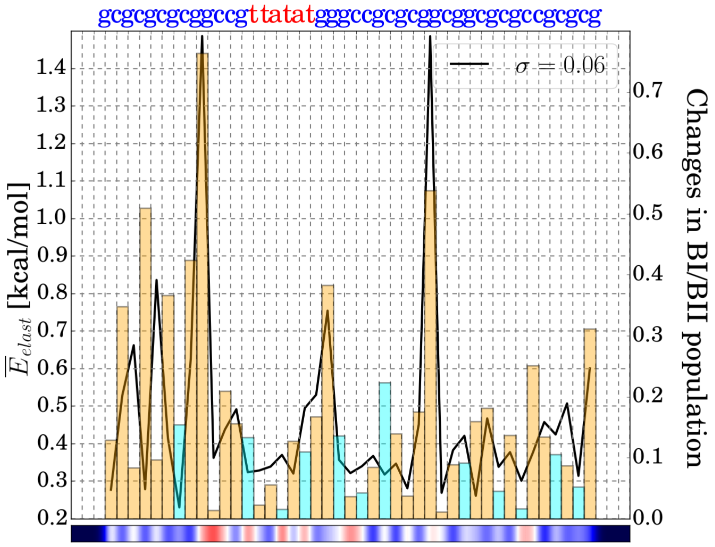

Figure S30: Average elastic energy for all central 43 base-pair steps for the gc-sequence simulated in the 9th Umbrella window. The plot was generated in the same way as Figure S22.

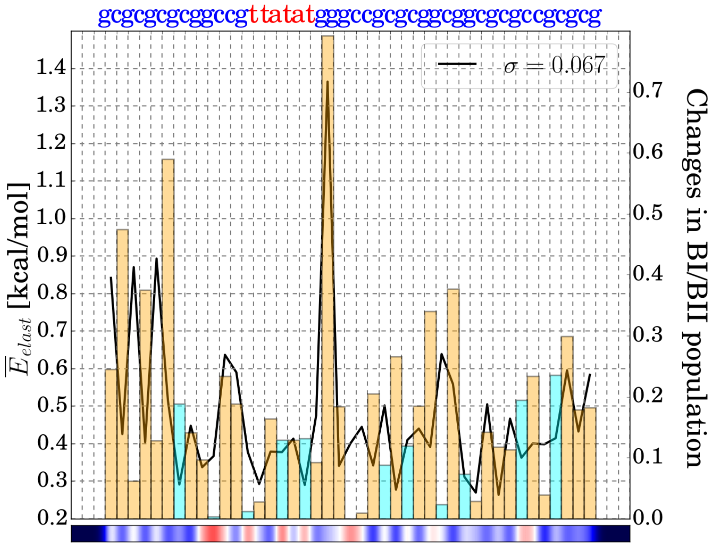

Figure S31: Average elastic energy for all central 43 base-pair steps for the gc-sequence simulated in the 10th Umbrella window. The plot was generated in the same way as Figure S22.

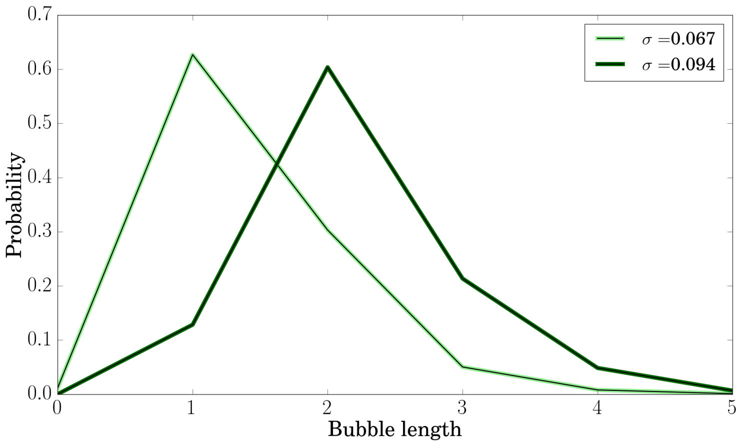

Figure S32: Probabilites for different sizes of the bubble length (number of denatured bps) as obtained from the Ising model, shown for two different levels of unwinding of the AT-sequence.

4 Ising model

In our implementation of the Ising model, we considered only macrostates with at most one denaturation bubble. Thereby, we iterate through all steps of the sequence as bubble-initiation steps and consider various bubble-lengths. We considered bubble sizes smaller than 6 molten base-pairs. Expected bubble-sizes for our system are quite short, $\sim 1 - 2bp$ (Figure S32).

5 Calculation of Geometric parameters through Rigid-Body Transformation

In order to quantify TATA-Box geometries, we set-up a new protocol, which allows us to calculate all base-pair and base-pair-step parameters. The major differences to other protocols (such as Curves+) are:

- All rotational parameters are Euler angles in our method (including also opening and buckle).
- We can easily obtain a reference-axis system for every base and base-pair. This also enables us to measure bending as the angle of the axis-vectors, which are orthogonal to the respective base-pairs.
- No specifications are required. Our script requires only topology- and trajectory files as input.

In the following, we outline our protocol:

First, we span a local reference system on every base. The local **x**-vector is the glycosidic bond. We then define a preliminar vector y_p as the vector-difference between the two base-carbon atoms next to the glycosidic nitrogen (e.g. C2, C6 in case of thymine). In the Crick-strand we define this vector in opposite direction. The local **z**-vector is obtained by: $\mathbf{z} = \mathbf{x} \times y_p$. We then calculate the actual **y**-vector through $\mathbf{y}=\mathbf{x} \times \mathbf{z}$. We place this axis-system, **A**, on the corresponding C1' atom, which we denote as anchor-points.

Second, we calculate the six intra base-pair parameters. The three translational parameters are given by the vectors connecting the anchor-points of paired bases. In a next step, we calculate the rotation matrix **R**, which transforms the Watson-Axis system into the Crick-Axis system: $\mathbf{R} = \mathbf{A}_{Crick}\ \mathbf{A}^T_{Watson}$. From this matrix, the three intra base-pair parameters are obtained as Euler-angles.

Third, we calculate the six base-pair-step parameters. For this, we first determine a mid-axis system for every base-pair by performing half-translation and half-rotation between the axis-systems of paired bases. The base-pair-step parameters follow from rigid-body-transformations between successive mid-axis systems analog to the calculation of intra base-pair parameters.

Bending angles are calculated as the angle between the **z**-vectors of the mid-axis system of the chosen base-pairs.

This procedure can be applied to consecutive base-pairs, but also to distant base-pairs. Our script runs efficiently and can be obtained upon request.
